# Supplementary material for: Interactions between donor Agreeableness and recipient characteristics in predicting charitable donation and positive social evaluation
Source: PeerJ. 2015 Aug 18;3:e1089. doi: 10.7717/peerj.1089 (PMC4548474; doi:10.7717/peerj.1089)

**Supplementary Table 1.** Sample dynamically generated stimuli.

Kirsten is addicted to painkillers. She feels trapped by her addiction. She helps at her spiritual center's weekly meditation-instruction groups. She canvasses for the Republican Party two hours per week.

Daniel has lung cancer. He only smoked cigarettes on weekends when he went out in high school and college. He attends church every Sunday. He has a reputation as somewhat of a gossip.

Anthony is paralyzed. He became paralyzed due to trauma from a tumor. He serves free vegetarian meals every Wednesday at his temple. He attends peace rallies once a month.

Latisha is a veteran with an amputated limb. She is now in a wheelchair. She did well in high school. She helps grow a community garden whose produce is for school lunches.

David has diabetes. He led an unhealthy lifestyle, which has resulted in the disorder. He helps grow a community garden whose produce is for school lunches. He often writes Letters to the Editor supporting the Green Party.

Emily has liver cancer. She drank heavily for many years. She reads the New York Times every morning. She loses her temper easily.

**Supplementary Table 2**. List of items participants rated for each target biography.

Sym1. I feel tender towards this person

Sym2. I don’t care about this person.

Sym3. I feel emotionally connected to this person

Dist1. I feel distressed by this person's suffering

Dist2. This person's story does not upset me

Dist3. I feel guilty about this person’s suffering

Sim1. My interests and hobbies are similar to this person's interests and hobbies

Sim2. My joys and fears are different from this person's joy and fears

Sim3. My personal morals and values are similar to this person's morals and values

Sim4. My economic status is similar to this person's economic status

Sim5. My level of education is different from this person's level of education

Sim6. My position in society is similar to this person's position in society

Need1. Having more money would not improve this person's situation

Need2. If charities helping people in this situation had more money, they would be able to do a lot of good

Need3. This person really needs help

Resp1. This person is responsible for their suffering

Resp2. I blame this person for their suffering

Resp3. This person's suffering is unjust or unfair

Like1. I am inspired by this person's story

Like2. I would like to meet this person in real life

Like3. This person does not seem trustworthy

Intent1. I would be very likely to help this person if I had the opportunity

Items are grouped by putative latent dimension (as determined in a pilot study in which no personality data were acquired). Sym = Sympathy; Dist = Distress; Sim = Self-similarity; Need = Neediness; Resp = Responsibility; Like = Likeability; Intent = Intention to help.

**Supplementary Table 3.** Person x situation effects on charitable behavior for Big Five traits.

| **NEUROTICISM** |  |  |  |  |
| --- | --- | --- | --- | --- |
|  | Norm prediction | Person x situation model | | |
| Dimension | Multiple R | norm | N | norm:N |
| Perceived responsibility | 0.71 | 1.12 (0.03)*** | 0.00 (0.04) | -0.05 (0.03) |
| Perceived likeability | 0.56 | 0.73 (0.03)*** | -0.01 (0.04) | -0.06 (0.03)* |
| Perceived neediness | 0.27 | 0.41 (0.02)*** | 0.02 (0.04) | -0.02 (0.02) |
| Felt sympathy | 0.46 | 0.56 (0.02)*** | 0.02 (0.05) | -0.06 (0.02)** |
| Felt distress | 0.42 | 0.53 (0.02)*** | 0.14 (0.05)* | -0.03 (0.02) |
| Intent to help | 0.39 | 0.68 (0.03)*** | -0.01 (0.07) | -0.04 (0.03) |
| Donation amount | 0.39 | 1.09 (0.06)*** | 0.06 (0.17) | -0.07 (0.06) |
|  |  |  |  |  |
| **EXTRAVERSION** |  |  |  |  |
|  | Norm prediction | Person x situation model | | |
| Dimension | Multiple R | norm | E | norm:E |
| Perceived responsibility | 0.71 | 1.12 (0.03)*** | -0.05 (0.04) | 0.04 (0.03) |
| Perceived likeability | 0.56 | 0.73 (0.03)*** | 0.15 (0.04)*** | 0.07 (0.03)** |
| Perceived neediness | 0.27 | 0.41 (0.02)*** | 0.08 (0.04). | -0.02 (0.02) |
| Felt sympathy | 0.46 | 0.56 (0.02)*** | 0.20 (0.05)*** | 0.05 (0.02)* |
| Felt distress | 0.42 | 0.53 (0.02)*** | 0.09 (0.05) | 0.04 (0.02) |
| Intent to help | 0.39 | 0.68 (0.03)*** | 0.22 (0.07)** | 0.01 (0.03) |
| Donation amount | 0.39 | 1.09 (0.06)*** | -0.03 (0.17) | -0.00 (0.06) |
|  |  |  |  |  |
| **OPENNESS** |  |  |  |  |
|  | Norm prediction | Person x situation model | | |
| Dimension | Multiple R | norm | O | norm:O |
| Perceived responsibility | 0.71 | 1.13 (0.03)*** | -0.21 (0.04)*** | 0.05 (0.03) |
| Perceived likeability | 0.56 | 0.73 (0.03)*** | 0.11 (0.04)* | 0.03 (0.03) |
| Perceived neediness | 0.27 | 0.41 (0.02)*** | 0.16 (0.04)*** | 0.04 (0.02)* |
| Felt sympathy | 0.46 | 0.56 (0.02)*** | 0.18 (0.05)*** | 0.01 (0.02) |
| Felt distress | 0.42 | 0.53 (0.02)*** | 0.17 (0.05)** | 0.01 (0.02) |
| Intent to help | 0.39 | 0.68 (0.03)*** | 0.32 (0.07)*** | -0.00 (0.03) |
| Donation amount | 0.39 | 1.10 (0.06)*** | 0.21 (0.17) | -0.00 (0.06) |

| **AGREEABLENESS** |  |  |  |  |
| --- | --- | --- | --- | --- |
|  | Norm prediction | Person x situation model | | |
| Dimension | Multiple R | norm | A | norm:A |
| Perceived responsibility | 0.71 | 1.12 (0.03)*** | -0.27 (0.04)*** | 0.06 (0.03). |
| Perceived likeability | 0.56 | 0.73 (0.03)*** | 0.20 (0.04)*** | 0.10 (0.03)*** |
| Perceived neediness | 0.27 | 0.41 (0.02)*** | 0.20 (0.04)*** | 0.05 (0.02)* |
| Felt sympathy | 0.46 | 0.55 (0.02)*** | 0.23 (0.05)*** | 0.05 (0.02)* |
| Felt distress | 0.42 | 0.52 (0.02)*** | 0.18 (0.05)*** | 0.07 (0.02)** |
| Intent to help | 0.39 | 0.68 (0.03)*** | 0.38 (0.07)*** | 0.07 (0.03)* |
| Donation amount | 0.39 | 1.09 (0.06)*** | 0.40 (0.17)* | 0.13 (0.06)* |

| **CONSCIENTIOUSNESS** |  |  |  |  |
| --- | --- | --- | --- | --- |
|  | Norm prediction | Person x situation model | | |
| Dimension | Multiple R | norm | C | norm:C |
| Perceived responsibility | 0.71 | 1.12 (0.03)*** | -0.01 (0.04) | 0.10 (0.03)** |
| Perceived likeability | 0.56 | 0.73 (0.03)*** | -0.07 (0.04) | 0.09 (0.03)** |
| Perceived neediness | 0.27 | 0.41 (0.02)*** | 0.01 (0.04) | 0.02 (0.02) |
| Felt sympathy | 0.46 | 0.56 (0.02)*** | -0.02 (0.05) | 0.07 (0.02)*** |
| Felt distress | 0.42 | 0.53 (0.02)*** | -0.07 (0.05) | 0.04 (0.02). |
| Intent to help | 0.39 | 0.68 (0.03)*** | 0.02 (0.07) | 0.06 (0.03)* |
| Donation amount | 0.39 | 1.09 (0.06)*** | -0.28 (0.17) | 0.10 (0.06). |

**Supplementary Table 4.** Effects of Agreeableness on ratings and donation when models include demographic and Big Five covariates.

|  | Norm prediction | Person x situation model | | | | | | | | | |
| --- | --- | --- | --- | --- | --- | --- | --- | --- | --- | --- | --- |
| Dimension | Multiple R | norm | sex(Male) | sex(Other) | age | C | E | N | O | A | norm:A |
| Responsibility | 0.71 | 1.13 (0.03)*** | 0.14 (0.08). | 0.43 (0.35) | -0.00 (0.00) | 0.00 (0.01) | 0.00 (0.01) | -0.03 (0.01). | -0.05 (0.02)** | -0.27 (0.05)*** | 0.06 (0.03). |
| Likeability | 0.56 | 0.74 (0.03)*** | -0.08 (0.10) | 0.51 (0.41) | 0.00 (0.00) | -0.06 (0.02)*** | 0.06 (0.02)*** | 0.02 (0.02) | -0.00 (0.02) | 0.22 (0.05)*** | 0.10 (0.03)*** |
| Neediness | 0.27 | 0.41 (0.02)*** | -0.03 (0.09) | 0.55 (0.37) | 0.00 (0.00) | -0.00 (0.01) | 0.02 (0.01) | 0.04 (0.01)* | 0.04 (0.02)* | 0.21 (0.05)*** | 0.05 (0.02)* |
| Sympathy | 0.45 | 0.56 (0.02)*** | -0.18 (0.12) | 0.21 (0.48) | -0.00 (0.00) | -0.04 (0.02). | 0.08 (0.02)*** | 0.05 (0.02)* | 0.01 (0.02) | 0.25 (0.06)*** | 0.05 (0.02)* |
| Distress | 0.44 | 0.53 (0.02)*** | -0.20 (0.12) | -0.30 (0.49) | 0.00 (0.00) | -0.02 (0.02) | 0.05 (0.02)* | 0.07 (0.02)*** | 0.01 (0.02) | 0.25 (0.06)*** | 0.07 (0.02)** |
| Intent to help | 0.39 | 0.68 (0.03)*** | -0.14 (0.16) | 0.67 (0.64) | 0.01 (0.01). | -0.02 (0.03) | 0.07 (0.03)* | 0.06 (0.03)* | 0.06 (0.03)* | 0.34 (0.08)*** | 0.07 (0.03)* |
| Donation amt | 0.4 | 1.10 (0.06)*** | 0.12 (0.37) | -1.11 (1.25) | 0.01 (0.01) | -0.16 (0.06)** | 0.03 (0.06) | 0.02 (0.06) | 0.06 (0.07) | 0.55 (0.19)** | 0.13 (0.06)* |

**Supplementary Table 5.** Person x situation effects on charitable behavior for each of the NEO-PI-R Agreeableness facets.

| **ALTRUISM** |  |  |  |  |
| --- | --- | --- | --- | --- |
|  | Norm prediction | Person x situation model | | |
| Dimension | Multiple R | norm | Altruism | norm:Altruism |
| Perceived responsibility | 0.71 | 1.12 (0.03)*** | -0.24 (0.04)*** | 0.09 (0.03)** |
| Perceived likeability | 0.56 | 0.73 (0.03)*** | 0.18 (0.04)*** | 0.12 (0.03)*** |
| Perceived neediness | 0.27 | 0.40 (0.02)*** | 0.20 (0.04)*** | 0.05 (0.02)* |
| Felt sympathy | 0.46 | 0.55 (0.02)*** | 0.25 (0.05)*** | 0.07 (0.02)** |
| Felt distress | 0.42 | 0.52 (0.02)*** | 0.19 (0.05)*** | 0.08 (0.02)*** |
| Intent to help | 0.39 | 0.68 (0.03)*** | 0.39 (0.07)*** | 0.08 (0.03)** |
| Donation amount | 0.39 | 1.09 (0.06)*** | 0.42 (0.17)* | 0.11 (0.06). |
|  |  |  |  |  |
| **TRUST** |  |  |  |  |
|  | Norm prediction | Person x situation model | | |
| Dimension | Multiple R | norm | Trust | norm:Trust |
| Perceived responsibility | 0.71 | 1.12 (0.03)*** | -0.15 (0.04)*** | 0.05 (0.03). |
| Perceived likeability | 0.56 | 0.73 (0.03)*** | 0.14 (0.04)** | 0.10 (0.03)*** |
| Perceived neediness | 0.27 | 0.40 (0.02)*** | 0.10 (0.04)* | 0.02 (0.02) |
| Felt sympathy | 0.46 | 0.55 (0.02)*** | 0.13 (0.05)* | 0.06 (0.02)** |
| Felt distress | 0.42 | 0.52 (0.02)*** | 0.02 (0.05) | 0.04 (0.02). |
| Intent to help | 0.39 | 0.68 (0.03)*** | 0.15 (0.07)* | 0.06 (0.03). |
| Donation amount | 0.39 | 1.09 (0.06)*** | 0.24 (0.17) | 0.07 (0.06) |
|  |  |  |  |  |
| **STRAIGHTFORWARDNESS** |  |  |  |  |
|  | Norm prediction | Person x situation model | | |
| Dimension | Multiple R | norm | Straight. | norm:Straight. |
| Perceived responsibility | 0.71 | 1.12 (0.03)*** | -0.24 (0.04)*** | 0.04 (0.03) |
| Perceived likeability | 0.56 | 0.73 (0.03)*** | 0.14 (0.04)** | 0.07 (0.03)** |
| Perceived neediness | 0.27 | 0.41 (0.02)*** | 0.15 (0.04)*** | 0.04 (0.02)* |
| Felt sympathy | 0.46 | 0.55 (0.02)*** | 0.20 (0.05)*** | 0.03 (0.02) |
| Felt distress | 0.42 | 0.52 (0.02)*** | 0.17 (0.05)** | 0.05 (0.02)* |
| Intent to help | 0.39 | 0.68 (0.03)*** | 0.31 (0.07)*** | 0.05 (0.03). |
| Donation amount | 0.39 | 1.09 (0.06)*** | 0.38 (0.17)* | 0.13 (0.06)* |
|  |  |  |  |  |
| **COMPLIANCE** |  |  |  |  |
|  | Norm prediction | Person x situation model | | |
| Dimension | Multiple R | norm | Compliance | norm:Compliance |
| Perceived responsibility | 0.71 | 1.12 (0.03)*** | -0.16 (0.04)*** | 0.02 (0.03) |
| Perceived likeability | 0.56 | 0.73 (0.03)*** | 0.17 (0.04)*** | 0.06 (0.03)* |
| Perceived neediness | 0.27 | 0.41 (0.02)*** | 0.13 (0.04)*** | 0.03 (0.02). |
| Felt sympathy | 0.46 | 0.55 (0.02)*** | 0.13 (0.05)* | 0.03 (0.02) |
| Felt distress | 0.42 | 0.52 (0.02)*** | 0.06 (0.05) | 0.03 (0.02) |
| Intent to help | 0.39 | 0.68 (0.03)*** | 0.26 (0.07)*** | 0.06 (0.03)* |
| Donation amount | 0.39 | 1.09 (0.06)*** | 0.19 (0.17) | 0.09 (0.06) |
|  |  |  |  |  |
| **MODESTY** |  |  |  |  |
|  | Norm prediction | Person x situation model | | |
| Dimension | Multiple R | norm | Modesty | norm:Modesty |
| Perceived responsibility | 0.71 | 1.12 (0.03)*** | -0.12 (0.04)** | 0.04 (0.03) |
| Perceived likeability | 0.56 | 0.73 (0.03)*** | -0.03 (0.04) | 0.02 (0.03) |
| Perceived neediness | 0.27 | 0.40 (0.02)*** | 0.06 (0.04) | 0.04 (0.02)* |
| Felt sympathy | 0.46 | 0.55 (0.02)*** | -0.08 (0.05) | 0.01 (0.02) |
| Felt distress | 0.42 | 0.52 (0.02)*** | 0.02 (0.05) | 0.05 (0.02)* |
| Intent to help | 0.39 | 0.68 (0.03)*** | 0.03 (0.07) | 0.04 (0.03) |
| Donation amount | 0.39 | 1.09 (0.06)*** | 0.10 (0.17) | 0.08 (0.06) |
|  |  |  |  |  |
| **TENDER-MINDEDNESS** |  |  |  |  |
|  | Norm prediction | Person x situation model | | |
| Dimension | Multiple R | norm | Tender. | norm:Tender. |
| Perceived responsibility | 0.71 | 1.12 (0.03)*** | -0.29 (0.04)*** | 0.02 (0.03) |
| Perceived likeability | 0.56 | 0.73 (0.03)*** | 0.28 (0.04)*** | 0.06 (0.03)* |
| Perceived neediness | 0.27 | 0.40 (0.02)*** | 0.24 (0.04)*** | 0.02 (0.02) |
| Felt sympathy | 0.46 | 0.55 (0.02)*** | 0.42 (0.05)*** | 0.02 (0.02) |
| Felt distress | 0.42 | 0.52 (0.02)*** | 0.36 (0.05)*** | 0.05 (0.02)* |
| Intent to help | 0.39 | 0.68 (0.03)*** | 0.57 (0.06)*** | 0.01 (0.03) |
| Donation amount | 0.39 | 1.09 (0.06)*** | 0.44 (0.17)** | 0.08 (0.06) |

**Supplementary Figure 1.** Subject-level fits for all rating dimensions. Colored lines reflect individual subject fits; black lines reflect the means for high-Agreeableness (> 1 SD from mean; solid line) and low-Agreeableness (< 1 SD from mean; dashed line) participants.


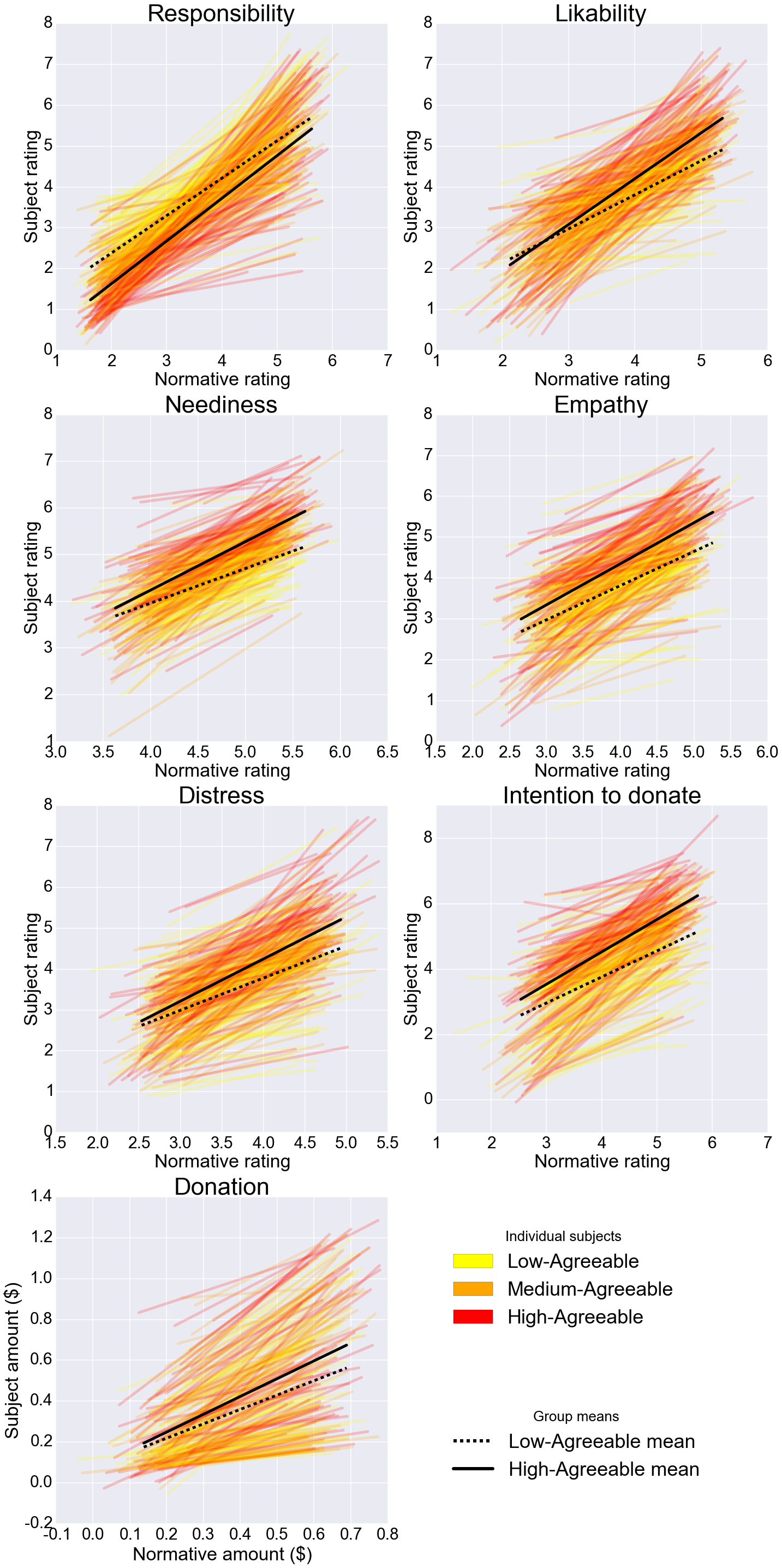

Supplement: Supplemental Information 1 [file peerj-03-1089-s001.docx]
